# Lifespan Extension in Long-Lived Vertebrates Rooted in Ecological Adaptation

**DOI:** 10.3389/fcell.2021.704966

**Published:** 2021-10-18

**Authors:** Olatunde Omotoso, Vadim N. Gladyshev, Xuming Zhou

**Affiliations:** ^1^CAS Key Laboratory of Animal Ecology and Conservation Biology, Institute of Zoology, Beijing, China; ^2^University of Chinese Academy of Sciences, Beijing, China; ^3^Division of Genetics, Department of Medicine, Brigham and Women’s Hospital and Harvard Medical School, Boston, MA, United States

**Keywords:** longevity, adaptive-hitchhiking, natural selection, aging, evolution theory

## Abstract

Contemporary studies on aging and longevity have largely overlooked the role that adaptation plays in lifespan variation across species. Emerging evidence indicates that the genetic signals of extended lifespan may be maintained by natural selection, suggesting that longevity could be a product of organismal adaptation. The mechanisms of adaptation in long-lived animals are believed to account for the modification of physiological function. Here, we first review recent progress in comparative biology of long-lived animals, together with the emergence of adaptive genetic factors that control longevity and disease resistance. We then propose that hitchhiking of adaptive genetic changes is the basis for lifespan changes and suggest ways to test this evolutionary model. As individual adaptive or adaptation-linked mutations/substitutions generate specific forms of longevity effects, the cumulative beneficial effect is largely nonrandom and is indirectly favored by natural selection. We consider this concept in light of other proposed theories of aging and integrate these disparate ideas into an adaptive evolutionary model, highlighting strategies in decoding genetic factors of lifespan control.

## Introduction

The aging process, also known as senescence, involves the gradual decline in vitality as a result of deterioration of physiological and biochemical functions, which subsequently leads to increased morbidity and mortality. Most of the organisms age, but some age slower than others (Finch, 1998), driving the fundamental question what underlying factors contribute to variation in aging processes across species, as well as to potential interventions to extend healthspan in humans (Reichard, 2016). Current research is hinged on the early theories of aging (Medawar, 1952; Harman, 1956; Williams, 1957; Hamilton, 1966) with supporting empirical evidence (Luckinbill et al., 1984; Austad, 1993; Shattuck and Williams, 2010). It is now well established that the mechanisms that control aging processes are under genetic regulation (Guarente and Kenyon, 2000; de Magalhães et al., 2007) and that these genetic regulators are constrained by environmental factors (Clare and Luckinbill, 1985; Williams and Day, 2003; Jobson et al., 2010). However, right from the onset, since the days of Charles Darwin, the evolvability of longevity, which means a duration of aging, has remained contentious, evolving alongside the concept of evolution (Gavrilov and Gavrilova, 2002). In the context of evolution by natural selection, longevity, but not aging, should be beneficial to species propagation; therefore, aging should have faded out to give way for immortal species. This is in contrast to the prevailing evidence that, while vertebrates eventually age and die, various species with genetic proximity, such as rodents, may have widely varying lifespans (Gorbunova et al., 2008). Disagreement still exists regarding the mechanisms that determine the variability of species’ lifespan across the tree of life (Jones et al., 2014), just as the process of aging remains controversial (Goldsmith, 2004; Kirkwood, 2005; Blagosklonny, 2013).

Despite the role that earlier evolutionary theories have played in experimental research relating to animal aging, these theories are delimited by the wide scope of aging and lifespan patterns observed under different environmental conditions across the tree of life (Jones et al., 2014). Subsequent models and empirical evidence have highlighted the shortcomings and shown that some of the general assumptions represent a drastically simplified version of a complex phenomenon (Abrams, 1993; Reznick et al., 2004; Caswell, 2007; Wensink et al., 2017; Moorad et al., 2019). For example, the classical evolutionary theories have assumed that lifespan evolves in response to the rate of extrinsic mortality, such that rapid senescence evolves under high extrinsic mortality. Conversely, it was reported that an increase in random extrinsic mortality does not evolve rapid senescence (Reznick et al., 2004; Wensink et al., 2014). More so, contrary to the assumptions on tradeoff (Williams, 1957; Kirkwood and Rose, 1991), the classical theories could not explain the unabated fecundity rate coupled with negligible senescence in turtles, tuatara, and other species (Cohen et al., 2020), and why some long-lived species show no sign of progressive aging as predicted by these theories (Vaupel et al., 2004; Finch, 2009; Ruby et al., 2018). There are indications that senescence, although rampant among complex species like mammals, might not be a universal phenomenon among all extant organisms (Jones et al., 2014). The paucity of empirical evidence to support the presence of biological tradeoff (Kirkwood and Holliday, 1979) between somatic maintenance and reproductive fitness indicates that tradeoffs cannot be the sole driver of aging or lifespan (Cohen et al., 2020). Another piece of evidence that does not support the disposable soma theory is found in human females that invest higher resources in reproduction yet outlive their male counterparts (Blagosklonny, 2010).

Interestingly at the moment, there is no explicit *a priori* theory to explain how natural selection works during evolution to extend lifespan, even though various studies on extremely long-lived animals try to uncover anti-aging mechanisms by searching for relevant genes and pathways under positive selection. In this regard, new hypotheses and models are required to better integrate and explain the findings. Here, we review adaptive responses that may drive longevity and propose an adaptive hitchhike model as a possible mechanism through which longevity evolves in the wild. We posit that lifespan extension is earned from adaptive changes embedded in animal genomes that control all life functions. Basic longevity criteria such as enhanced immune functions, efficient DNA response and repair, tight control of the cell cycle, and genome maintenance are shared among all long-lived species and are under adaptive regulation, but the major contributing factors are species-specific adaptations to their respective ecological niche, especially under extreme environments.

## Crosstalk Between Adaptation and Lifespan Extension

The ability of an organism to survive in a specific ecosystem as a result of changes to its behavioral, physiological, morphological, and genetic response is called adaptation. Ecological adaptation strongly underlies lifespan extension in lineages and species where longevity has been observed despite differences in species ecosystem, morphology, and complexity. Predictably, all long-lived species have low extrinsic mortality due to the nature of their habitat or have evolved mechanisms to evade predators and imminent dangers (Harvey and Purvis, 1999). For example, while bats and birds have evolved powered flight to search for food and escape from predators and unfavorable weather conditions, thereby reducing extrinsic mortality (Munshi-South and Wilkinson, 2010), mole-rats are subterranean and have even fewer predators (Lewis and Buffenstein, 2016), reptiles have a scaly shell to guard against predators and hazardous environments, and species like elephants, cetaceans, and other large animals have few predators due to their body size. Humans are highly intelligent and thereby have also developed a protected environment that contributes to lifespan extension (Austad, 1997). However, as much as this ability is expected to contribute to lowering extrinsic mortality, it neither explains the variation observed in lifespan or mechanisms through which lifespan is regulated.

**FIGURE 1:**
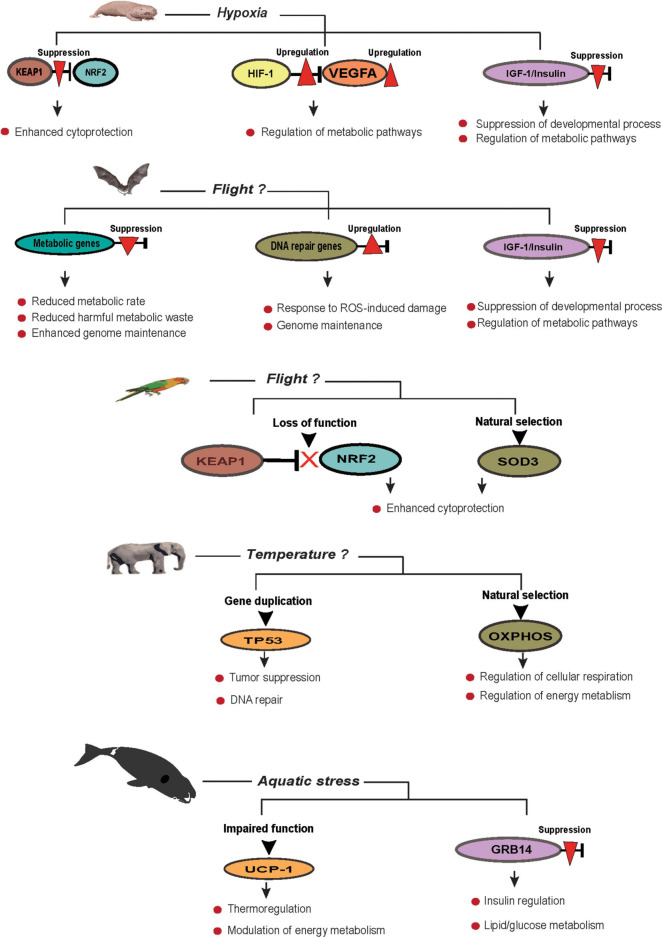
Environmental and morphological contributions to an extended lifespan.

Long-lived species are now known to exhibit efficient adaptive responses in essential pathways that contribute to lifespan with evidence of enhanced genome maintenance, DNA damage response, and repair attributing to their longevity (Gorbunova et al., 2014; Tian et al., 2019). Thanks to affordable genome sequencing, the availability of genome data revealed widespread adaptation in the genomes of long-lived species where positive selection, rare sequence variants, and genome duplication contributed to ecological adaptation, the evolution of body size, and disease resistance (Abegglen et al., 2015; Huang et al., 2019; Zhou et al., 2020). Although mechanisms of extended lifespan of these species are currently unclear, emerging evidence from genomic analyses points to the important role of species adaptation in longevity (Figure 1). This section reviews organismal adaptations across different taxa and highlights how natural selection contributed to the longevity of these species through adaptive responses to different ecological niches.

### Hypoxic Adaptation Potentially Promotes Longevity

While various environments may support basic adaptive survival, some ecosystems have stringent features that limit the species that could survive in such habitats; examples include hot springs, the Arctic, and high mountains. Organisms that live in an environment with limited oxygen (hypoxic conditions), for instance, face persistent challenges of hypoxia and must develop physiological features that would aid in their survival in such a challenging environment. Evolutionary adaptations have largely converged across these species to address oxygen demand either by optimizing oxygen uptake or reducing oxygen requirement for metabolism (Pamenter et al., 2020). Hypoxia-driven adaptation involves selection acting on pathways such as central metabolism, cellular respiration, hemoglobin-mediated oxygen transport, and hypoxia-inducible factor pathways (Simonson, 2015; Ding et al., 2018), whereas other adaptive mechanisms in these species could be attributed to species physiology, ecological differences, lifestyle, and adaptive fitness (Pamenter et al., 2020).

The Tibetan population on the Tibet plateau is an example of a human population that has adapted to hypoxia conditions at high altitudes (Beall et al., 2010); conversely, naked mole-rats are adapted to hypoxic conditions in a subterranean niche (Fang et al., 2014b), while species such as turtles and cetaceans (e.g., bowhead whale) have adapted to intermittent hypoxia conditions (Keane et al., 2015; Tian et al., 2016). Hypoxic adaptation is believed to contribute to longevity in these organisms as incidences of hypoxia have been reported to extend lifespan from invertebrates to vertebrate animals (Mehta et al., 2009; Boretto et al., 2018). Hypoxia-inducible factor (HIF-1) is the chief mediator of hypoxia-response pathways that are usually activated in low-oxygen conditions, and modulation of this pathway and HIF-1 has resulted in varying degrees of longevity (Leiser et al., 2013). HIF-1 has, however, been strongly linked to different types of tumor progression and its constitutive expression mediates intra-tumoral hypoxia (Zhong et al., 1999; Semenza, 2010). Nevertheless, HIF-1 and the hypoxia-response pathway have been touted as a viable intervention for lifespan extension as demonstrated in *Caenorhabditis elegans* (Mehta et al., 2009; Leiser et al., 2013). In a recent study, mice exposed to chronic hypoxia [11.8% atmospheric (atm) O_2_] for 32 days demonstrated gene expression patterns that were similar to known longevity interventions including calorie restriction (Tyshkovskiy et al., 2019); this suggests that the mechanisms of lifespan control might have converged on related genes and pathways and exhibit a similar pattern of behavior when modulated by longevity interventions (Tyshkovskiy et al., 2019). Hypoxia is likely able to counteract and redeem the detrimental effects associated with aging-related pathways. As recently discovered, centenarians on the Tibet Plateau have a longer lifespan compared with any other region in China (Li et al., 2017). Genetic variation indicating rapid evolution, was significantly higher in aging-related genes compared to other genes in Tibetans than in the Han population; this study further found a significant negative association between expressed genes under hypoxia and during aging (Li et al., 2017). Similar to hypoxia-induced longevity in model organisms, hypoxia conditions upregulate longevity-associated genes that are usually downregulated during aging (Kim et al., 2011; Li et al., 2017). As expected, hypoxia-related genes and pathways are under strong selection on the Tibet plateau (Yi et al., 2010; Xu et al., 2011; Basang et al., 2015). The events leading to the acquisition of this unique adaptation are sketchy, but it has been speculated that adaptation to high altitude among Tibetans might have been transferred through introgression from the now-extinct *Homo denisova* (Denisovans) that inhabited the plateau during the mid-Pleistocene, circa 50,000 years ago (Huerta-Sánchez et al., 2014; Chen et al., 2019).

In the naked mole-rat (NMR), adaptation to underground hypoxia has been one of the central focuses in studying molecular mechanisms that support its longevity. Naked mole-rats and other subterranean mole-rats with extended lifespan share a similar pattern of adaptive constraint in their genomes and physiological architecture that have aided adaptation to underground burrows in contrast to non-subterranean mammals (Bennett and Faulkes, 2000; LaVinka et al., 2009). For instance, sweeping changes were observed in the amino acid residues of arginase 1 (ARG1), which functions in the urea cycle, and increased ARG1 activity has been linked to certain pathologies including human cancer (Fang et al., 2014b; Caldwell et al., 2018). Higher expression of hypoxia-related genes, DNA repair, and globin proteins was observed in all subterranean mole-rats as well as highland inhabitants that were evaluated for hypoxia adaptation (Fang et al., 2014a, b). Hypoxia adaptation induces hypothermia and hypo-metabolism, which are physiological hallmarks similar to hibernating species and CR animals, and with links to extended lifespan (Lewis et al., 2018). Likewise, mice exposed to a model of NMR natural environment (hypoxic–hypercapnic environment) exhibit a significant decrease in both body temperature and metabolic rate as observed in CR and mutant mice (Conti et al., 2006; Tolstun et al., 2020). There were no significant expression changes in stress-related genes, while also displaying accelerated wound healing (Tolstun et al., 2020).

Experimental manipulation of adaptive mechanisms in key aging-related pathways has led to similar lifespan extension, although sometimes with negative pleiotropic or epistasis effects in model organisms (Mehta et al., 2009). Hypoxia studies in the rotifer *Brachionus manjavacas*, an aquatic invertebrate suitable for aging studies, demonstrated a mean lifespan extension of 107% when continuously exposed to 1.6% atm. Hypoxic conditions in this organism increased reproductive success by twofold and conferred cytoprotection against stress (Snell et al., 2019). This observation supports previous studies on rotifer communities and their adaptation to high salinity and low oxygen (Esparcia et al., 1989). In *C. elegans*, lifespan extension was reported in both wild and mutant animals exposed to 1% atm. O_2_ (Honda et al., 1993), while in Drosophila, adult lifespan was extended under moderate hypoxia conditions (10 kPa) (Rascón and Harrison, 2010). Hypoxia-induced stress in *C. elegans* has been linked to lifespan extension *via* the modulation of the HIF-1 pathway (Leiser et al., 2013), and loss of function of VHL-1 (von Hippel–Lindau 1), which negatively regulates HIF-1, resulted in a significant increase in lifespan in *C. elegans* (Mehta et al., 2009). Under different dietary restriction regimens, however, chronic hypoxia and HIF1 activity caused a reduction in lifespan in Drosophila and *C. elegans*, respectively (Vigne and Frelin, 2006; Chen et al., 2009). As a caveat, the hypoxic response in hypoxia-sensitive species such as many model organisms would be expected to differ from those of hypoxia-tolerant organisms adapted to a hypoxic habitat in the wild. Yet, a formal phylogenetic analysis is needed to evaluate the pro-longevity response of hypoxia. Altogether, these observations highlight the importance of HIF-1 and the hypoxia-response pathway to modulate lifespan. We infer from the efficient cancer resistance mechanisms in the NMR (Seluanov et al., 2018) that adaptation to hypoxic conditions contributes to the suppression of the pro-cancer activities of HIF-1 and the hypoxia-response pathway, thereby extending lifespan. Overall, hypoxia adaptation contributes to longevity, although in a complex manner (Chen et al., 2009; Zhou et al., 2011; Leiser et al., 2013), as observed in the Tibetan population, mole-rats, and various model organisms.

### Aquatic Lifestyle-Associated Adaptation and Lifespan Extension

Similar to the hypoxic terrestrial environment, the Arctic and Antarctic regions are highly unfavorable for many organisms but house some of the longest-lived vertebrates on Earth. The Arctic is characterized by extremely low temperatures, salinity, and limited resources, which greatly influence the type of animals that could adapt to such a challenging environment (Gradinger, 2001). Animals in this region have evolved physiological, behavioral, and physical adaptations to cope with this extreme condition (Blix, 2016). Species such as bowhead whales (*Balaena mysticetus*, BHW) and Greenland sharks (*Somniosus microcephalus*) are among the longest-lived vertebrates with a maximum lifespan of over 200 years, with the latter speculated to be capable of reaching an age above 300 years (George et al., 1999; Nielsen et al., 2016). These species have been poorly studied for their longevity trait, but adaptation, mainly driven by temperature, diet, and metabolism, has contributed to an extended lifespan in cetaceans (Keane et al., 2015).

Large body size in these species evolved alongside enhanced cancer defense mechanisms, efficient tumor suppressors, translating to a lifetime of low cancer risk; this indicates a strong interplay between molecular mechanisms that underlie tumor suppression, large body size, and longevity (Caulin and Maley, 2011; Caulin et al., 2015; Nunney et al., 2015; Wensink, 2016). This large body size in cetaceans is maintained under a strong aquatic selection driven by thermoregulation, feeding efficiency, and metabolic rate (Gearty et al., 2018; Goldbogen et al., 2019). BHWs are filter feeders with access to abundant small prey and lipid-rich diet; they possess exceptionally high deposits of adipose tissue (blubber) and high leptin accumulation (Ball et al., 2017; Jiménez-Cortegana et al., 2021). This could partly explain the evolution of extreme large body size and low cancer in this species (Goldbogen et al., 2019). Both Greenland sharks and BHW experience intermittent episodes of hypoxia during deep diving, and adaptation to such low oxygen conditions has been attributed to extended lifespan in terrestrial species. The Greenland shark, just like all other long-lived species, lacks exceptional antioxidant protection (Costantini et al., 2017) but has an extremely low metabolic rate, as well as low daily energy demands (Ste-Marie et al., 2020). BHW genome encompasses adaptive traits that support extended lifespan and has been perpetuated through gene duplication and selection, while also pruning diseases and cancer susceptibility genes through pseudogenization and total gene loss. This aligns with recent reports of positive selection and widespread gene duplication in tumor suppressor genes in cetaceans; the large body size in this clade appears to correlate with strong selection in tumor suppression pathways (Tollis et al., 2019; Tejada-Martinez et al., 2021). These genes are implicated in a wide range of tumor types, metabolism, cell proliferation, and pathways believed to regulate animal longevity.

Evidence from genome sequencing and comparative genome analyses revealed several unique amino acid replacements in the bowhead whale genome that are associated with ecological adaptations, disease resistance, and cancer (Keane et al., 2015). For instance, *ERCC1* and *HDAC1* genes possess unique amino acid residues, while *PCNA* might have undergone possible selection in bowhead whales; these genes are linked to DNA repair and chromatin structure regulation pathways, respectively (Gillet and Schärer, 2006). More so, a lineage-specific mutation was observed in uncoupling protein 1 (*UCP1*) gene in the bowhead whale that is likely to contribute to molecular adaptation in thermoregulation and energy metabolism as previously observed in the naked mole-rat (Kim et al., 2011; Keane et al., 2015). Several genes that mediate in metabolism and hypoxia-tolerance pathways, including insulin and mTOR, are under adaptive selection in cetacean lineages (Tian et al., 2016; Nam et al., 2017; Derous et al., 2021); these are essential pathways for extended lifespan and play important roles in metabolism and ecological adaptation.

Compared with other mammals, differentially expressed genes (DEGs) in the bowhead whale liver support enhanced gluconeogenesis as an adaptive mechanism to high lipid diet (Berge et al., 2012). Downregulation of *GRB14* gene impacts insulin/IGF1 signaling pathway function and also serves as a shield against diet-related chronic diseases, while higher expression of *CITED2* gene mimics that of calorie restriction (Carré et al., 2008; Sakai et al., 2012), suggesting that these genes contribute to lifespan regulation through lipid and glucose metabolism (Wang et al., 2014). Liver DEGs also support elevated tolerance to hypoxia and enhanced DNA repair that could be considered adaptive mechanisms to the Arctic habitat (Seim et al., 2014). Furthermore, 53 DEGs were observed in the kidney, whose functions involve maintenance of genome integrity and tumor suppression, which may confer protection against age-related changes in the BHW. Unique amino acid changes in several proteins were also identified as well as evidence of positive selection in essential genes with roles in cancer, e.g., *MTUS1*, *GSK3A*, *PRUNE*, and *CYFIP1* (Seim et al., 2014; Ma and Gladyshev, 2017). Overall, longevity of these aquatic species is coherent with ecological adaptation driven by selective constraints acting on body size, genome, and species physiology.

### Metabolic Adjustment and Lifespan Extension

Aves are a successful class of vertebrates with ubiquitous longevity traits and an average life expectancy that supersedes that of most terrestrial mammals of comparable size (although few exceptions exist). According to the evolutionary theory of aging, their ability to fly shielded them from non-aging-related mortality (Pomeroy, 1990), hence, leading to longevity (Austad and Fischer, 1991). There seems to be no clear-cut adaptive mechanism that could be solely responsible for a long lifespan of birds, but the ability to fly and glide, and arboreal living in this class and other classes of vertebrates is believed to contribute to their longevity (Partridge and Barton, 1993; Holmes and Austad, 1994; Moorad and Promislow, 2010; Shattuck and Williams, 2010), although cytoprotective adaptation appears to protect birds from tissue damage due to metabolic waste (Holmes et al., 2001). Powered flight supports longevity of extant flying birds by aiding quick escape from predators, disease, famine, and other environmental hazards, just as in the case of birds, which evolved biochemical adaptations to cope with intense metabolic rate during flight and also repress the damaging consequences of metabolic by-products (Munshi-South and Wilkinson, 2010). Longevity attributes in birds include social organization and cognitive abilities as seen in parrots (Wirthlin et al., 2018), and slow senescence alongside high reproductive success at an advanced age (Partridge and Barton, 1993). Hummingbirds, parrots, seabirds, and songbirds are a few of the species that have independently evolved longevity through unique and shared adaptive traits (Holmes and Austad, 1995).

Similar to birds, bats are capable of powered flight and may be considered the longest living mammals when adjusted by body mass; the maximum lifespan of bats can be 3.5 times higher than their non-flying mammalian counterparts (Wilkinson and South, 2002; Wilkinson and Adams, 2019). With powered flight and being the only mammals that can do this, bats have been able to reduce extrinsic mortality by flying away from hazardous environments and predators (Pomeroy, 1990; Partridge and Barton, 1993). Other adaptations that contribute to bat longevity include hibernation, cave roosting, and torpor (Jürgens and Prothero, 1987; Wilkinson and South, 2002). Comparative studies revealed that extreme longevity has evolved among bats at least four times, and these four groups include the *Myotis*, horseshoe bats (*Rhinolophus*), long-eared bats (*Plecotus*), and vampire bats (*Desmodus rotundus*). All these lineages also possess the ability to hibernate, except for vampire bats that can undergo torpor at feeding intervals (McNab, 1969; Wilkinson and Adams, 2019). Vampire bats also have cooperative social behavior and a food sharing pattern that reduces the likelihood of starvation of roost mates that are unable to secure blood meals (Carter and Wilkinson, 2013; Carter et al., 2017). The role of hibernation in longevity in chiropterans has been controversial. While Herreid (1964) reported no observable differences in longevity between hibernating and non-hibernating bats, Wilkinson and South (2002) reported a significant increase in lifespan upon hibernation. However, both hibernating and non-hibernating species can live much longer than other mammals of similar body size (Wilkinson and Adams, 2019). Hibernation reduces the risk of mortality by predation and the risk of starvation; it also lowers metabolic rate and decreases body temperature (Nagel and Nagel, 1991; Wilkinson and South, 2002; Wilkinson and Adams, 2019). A reduction in the metabolic rate during hibernation is also expected to reduce the accumulation of metabolic waste that could induce cellular damage. Furthermore, physiological manifestations of hibernation in bats are similar to those produced by calorie restriction in rodents, which include a decrease in blood glucose and insulin, reduced glycolytic enzyme activity, increased protein synthesis, and strong antioxidant defenses (Walford and Spindler, 1997; Masoro, 2000).

Flight is the main reason for a high metabolic rate in bats and birds (Munshi-South and Wilkinson, 2010). In itself, flight is an efficient but costly strategy compared with other forms of locomotion in vertebrates (Norberg, 2012). Flight requires an extremely high mass-adjusted metabolic rate that is three to five times higher than in non-flying mammals, making flight a high energy-demanding trait (Speakman and Thomas, 2003). With this high metabolic rate, it may be expected that bats and birds would exhibit higher mutation rates and accordingly be short lived (Goyns, 2002; Ruf and Geiser, 2015). The ability of some bat species to support daily reduction in metabolic rate, i.e., enter the state of torpor, could greatly contribute to bat longevity. Small-bodied bat species are mostly found in the temperate zone and can maintain a near-absolute (99%) reduction in metabolic rate in a low-energy torpor state (Ruf and Geiser, 2015). Bats also possess the ability to conserve energy expended by lowering heart rates from a high 900 beats per minute during flight to as low as 200 beats per minute in a resting state, thus, reducing daily energy consumption by 10% (O’Mara et al., 2017). Reactive oxygen species-induced oxidative damage due to high metabolic rate might be a contributory factor to aging, but it has failed to explain longevity in birds and bats (Brunet-Rossinni, 2004; Montgomery et al., 2012; Xia and Møller, 2018).

Unique segmental deletion and reduced intergenic regions in birds, and also in bats, support gene proximity in the genomes, aiding in rapid expression and regulation of discrete sets of genes needed for metabolism during flight (Zhang and Edwards, 2012). Furthermore, the possibility of gene coevolution, as well as co-regulation, exists between power flight and metabolism in birds as multiple genes linked with each mechanism were found to be under positive selection (Zhang et al., 2014). The mechanisms by which birds and bats evade the deleterious effect, which is the price of a high metabolic rate is not fully understood. NRF2 gene mediates antioxidant response in metazoans by activating cascades of genes responsible for cytoprotection and metabolic functions (Yamamoto et al., 2018). In the absence of oxidative stress, KEAP1 targets NRF2 for degradation and maintains a low expression level of NRF2 in the cell. Interestingly, KEAP1 gene, which is conserved across metazoans, has undergone fragmentation in the lineage leading to the Neoaves (modern birds). KEAP1 loss of function means that Neoaves NRF2 cannot be targeted for degradation, leading to its constitutive expression in birds. This is thought to be an adaptive mechanism that shields cells and tissues from ROS-induced damage during increased metabolism (Castiglione et al., 2020). Similarly, overexpression of NRF2 and knock-down of KEAP1 increased the lifespan in *C. elegans* and *Drosophila*, respectively (Sykiotis and Bohmann, 2008; Tullet et al., 2017), while xenobiotic-metabolizing enzymes, which are downstream targets of NRF2, were upregulated following CR intervention in mice (Tyshkovskiy et al., 2019). Several genes and pathways associated with DNA damage and repair, cell cycle regulation, and metabolic pathways were found to be under selection pressure in parrots, red-crowned cranes, and other long-lived birds (Lee et al., 2020). Interestingly, *SOD3* gene was found to be under positive selection in parrots, red-crowned crane, ostrich, and other long-lived birds (Wirthlin et al., 2018; Lee et al., 2020) suggesting that birds may have evolved a similar adaptive response to combat the cost of high metabolic rates. However, regardless of species-specific metabolic rates, animals with extended longevity usually have a low generation rate of ROS in the mitochondria (Barja, 1999).

### Temperature-Driven Changes and Lifespan Extension

Reptiles are generally long-lived, comprising species that exhibit negligible senescence in the wild, and have adapted to a range of environments. Tuatara (*Sphenodon punctatus*) is a species of reptiles with extreme longevity coupled with reproductive success that could live far beyond 100 years. This may be the species with the lowest growth rate among reptiles (Cree, 2014). The optimal body temperature of the tuatara is between 16 and 21°C, and the animal remains active at temperatures below 5°C. This unusual temperature adaptation is under the control of the thermoregulatory genes called transient receptor potential (TRP) ion channels (Nilius and Owsianik, 2011). Thirty-seven gene copies of TRP were identified in the tuatara, many of which are under positive selection (Gemmell et al., 2020). Also, the tuatara has 26 selenoprotein genes, one more than humans, and 4 copies of selenocysteine-specific tRNA; these genes are responsible for redox regulation and support other functions. Adaptation to extremely low temperatures, protection against oxidative damage and disease resistance appeared to contribute to tuatara’s longevity (Gemmell et al., 2020).

Turtles are another reptile species that have been studied for negligible senescence, and accumulating empirical evidence revealed their longevity, with an unabated fecundity rate at an advanced age. Species such as Blanding’s turtle (*Emydoidea blandingii*), three-toed box turtle (*Terrapene Carolina triunguis*), and the painted turtles (*Chrysemys picta*) have been reported for their reproductive success even at advanced ages (Congdon et al., 2001, 2003; Miller, 2001). Longevity in turtles encompasses adaptive mechanisms against extreme conditions more than any terrestrial vertebrate and could barely be compared with naked mole rats. For instance, the pond slider (*Trachemys scripta*) can survive harsh cold weather conditions in complete absence of oxygen and can rely entirely on anaerobic glycolysis for many weeks (Lutz et al., 2003). Brain mitochondria of *Trachemys scripta* after 2 weeks of exposure to anoxia exhibit elevated tolerance to chronic anoxia, indicating that turtles evolved efficient endogenous mechanisms to support a switch between normoxia and anoxia conditions to regulate metabolic functions (Pamenter et al., 2016; Bundgaard et al., 2019). *Trachemys scripta* deploy elevated levels of cytoprotective proteins in the tissue, are highly stress-resistant, and maintain hypometabolism (Willmore and Storey, 1997; Krivoruchko and Storey, 2010); more so, turtles maintain a higher brain concentration of ascorbic acid that doubles what is present in the mammalian brain cortex (Rice et al., 1995). These extraordinary mechanisms of anoxia tolerance possibly play a crucial role in the species longevity as it is a common phenomenon for such animals to acquire adaptive traits to overcome ecological constraints (Söti and Csermely, 2007).

Thermoregulation is an integral part of animal metabolism. However, adaptive mechanisms that modulate thermoregulation and metabolism in elephants are not yet understood, neither they have been assessed for a possible link to elephant longevity and aging. However, what we know is that elephants live in a hot climate where the environmental temperature often exceeds the body temperature (Mole et al., 2016). With sweat glands lacking, one of the heat loss mechanisms in this species is the adaptive behavior of flapping large ears, but the genetic mechanism of heat transfer is poorly understood (Wright and Luck, 1984; Robertshaw, 2006). Nevertheless, elephants might have evolved adaptive temperature-sensing mechanisms to cope with habitat thermodynamics through ion channel TRPM8, also known as transient receptor potential melastatin 8 that takes part in temperature-gating mechanisms. A single point mutation at site 919 within the pore domain of TRPM8 of the elephant has been suggested to either be a modulatory site for temperature sensors or include synchronously dispersed temperature-sensing residues (Yang et al., 2020). Previous studies have identified diurnal heat storage mechanisms that are responsible for the regulation of daily heat fluctuation as a major thermoregulatory mechanism in large animals including elephants. This mechanism, also known as heterothermy, is common among large desert animals such as the camel and Arabian oryx, and it is an adaptive strategy allowing conservation of both energy and water in a hot and dry climate (Elder and Rodgers, 1975; Ostrowski and Williams, 2006; Weissenböck et al., 2012). Similar heterothermic behavior is an adaptive trait in bat lineages where the *leptin* gene that plays an essential role in thermogenesis and energy metabolism is under positive selection (Yuan et al., 2011). However, unlike bats, whether adaptive thermoregulation in elephants has a role in aging and extended lifespan is an open question. Evidence of adaptive evolution was detected in the electron transfer chain (ETC) complex in the African elephants; two genes that encode NADH dehydrogenase and ATP synthase, and function in the oxidative phosphorylation pathway (OXPHOS) are under positive selection (Finch et al., 2014). Oxidative phosphorylation genes are responsible for energy metabolism, ATP generation, and heat production that are required for cellular activities.

Evolutionary events that led to gene duplication of *TP53* gene have been described in elephants; African elephants have 19 extra copies of *TP53* retrogenes (pseudogenes) (*TP53RTG1-19*) with evidence of expression (Abegglen et al., 2015; Sulak et al., 2016). It was suggested that these pseudogenes support lower cancer occurrence in these species, although more research is needed to establish whether this is the case (Caulin and Maley, 2011; Abegglen et al., 2015). One possible explanation is the protagonist pleiotropy, wherein longevity traits could be driven by selection against cancer-related damage (de Grey, 2007). Thus, one of the factors contributing to extended lifespan in the elephant may be the strong selection acting on tumor suppressor genes. Beyond the canonical anticancer activities of *TP53* protein, cancer-unrelated functions of this protein have not been investigated in elephants. *TP53* takes part in several biological processes that include stress response, senescence, cell cycle regulation, insulin homeostasis, and cellular metabolism (Krstic et al., 2018). Owing to a wide range of functions of *TP53*, and its emerging role in cellular metabolism (Ranjan and Iwakuma, 2018), it is plausible to hypothesize that p53 duplication could have an adaptive role in elephant metabolism, thermoregulation, and habitat adaptation.

## The Adaptive-Hitchhike Model

**FIGURE 2:**
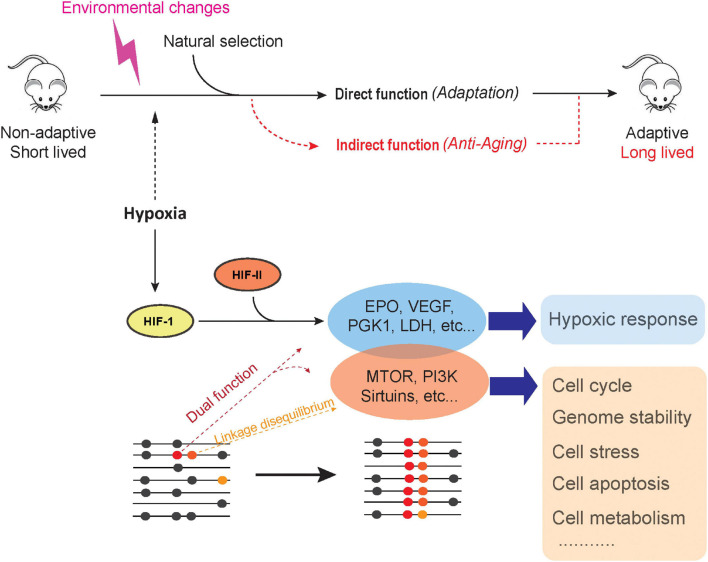
Adaptive-hitchhike model of animal longevity.

Although we discussed a number of genetic adaptions in the wild that contribute to lifespan extension, population genetics postulates that genetic changes are hardly to be fixed if such genetic changes do not increase fitness during long-term evolution. Nevertheless, we posit that strong selection acts to maintain these changes, leading to long lifespans in living organisms. We propose that extended lifespan is not by itself under selection but rather an epiphenomenon (by-product) of species adaptation, a phenomenon we have termed here the adaptive hitchhike model. First, the model implies that a new pleiotropic mutation, with one of its effects being extended lifespan, could be favored by natural selection due to its advantage to some other trait and therefore becomes fixed. Second, the model also applies to new pro-longevity mutations that occur at sites closely (or functionally) linked with the allelic sites under selection; if a new pro-longevity mutation arises at a site that is linked to an adapted genome region, natural selection may cause an increase in allele frequency and fix this pro-longevity mutation through linkage and allelic associations. Therefore, the adaptive-hitchhike model suggests that the selective constraint acting on the genomic region associated with adaptation and fitness is largely responsible for non-random beneficial pro-longevity effects. For example, patterns of selective sweep across loci of close proximities were reported for adaptation to altitude among the Tibetan population (Yi et al., 2010), and a further association was found between longevity and hypoxia response in this same population (Li et al., 2017). In other cases, natural selection might act on an already existing but neutral mutation through a sweeping selection; therefore, if neutral alleles responsible for lifespan extension are close enough to other alleles under selection, the chances of recombination are slim, and together, they become fixed in the population. This model could be mainly summarized in the following ways (Figure 2): (1) Some adaptive genetic changes could have dual functions, i.e., adaptive and longevity effects. (2) A pro-longevity mutation could come under selection and become fixed through direct selection or linkage and allelic association. (3) In the same way that a pro-longevity mutation could become fixed, a geronic (pro-aging) mutation could also become fixed and lead to a shorter lifespan. (4) In the case where environmental pressure is relaxed, pro-longevity effects may be lost. Therefore, our adaptive-hitchhike model of longevity of animals could be tested by (a) identifying pro-longevity effects of genetic changes that respond to adaptation and (b) detecting signals of linkage disequilibrium (LD) between adaptive and longevity variations. The novelty of this model is that it gives a key role to such nucleotide substitutions and loci with dual functions. Functional evaluation and validation of adaptive nucleotide substitutions with the pro-longevity potential could provide answers to the century-long questions surrounding the evolvability of animal lifespan.

## Relationship of the Model to Current Understanding of Aging

### Programmed Aging and Quasi-Programmed Aging

Programmed cellular aging, by analogy to *mitoptosis*, *apoptosis*, and *organoptosis*, which work at the level of mitochondria, cells, and tissues, respectively, involves an event of self-elimination (Skulachev and Longo, 2005). The quasi-programmed model on the other hand posits that damage (aging or senescence) is the later life unintentional consequence of early developmental programs because these programs would not turn off when development is completed. The argument is that the same pathways that drive soma development during early life are responsible for aging and cellular senescence; this argument is supported by an example of the mTOR/insulin signaling pathway that supports development but contributes to accelerated aging and could be downregulated to extend the lifespan (Blagosklonny, 2006, 2013; Wu et al., 2013). The quasi-programmed model partially explains the antagonistic pleiotropy theory of aging such wherein the consequences of later-age hyperfunction stem from early developmental processes, and therefore, the aging process appears programmed (but is not really programmed, i.e., there are no genes that evolved with the goal to cause aging). Both models emphasize the genetic determinism of the aging process, wherein programmed aging and quasi-programmed models argue about purposeful and indirect effects, respectively. We posit that longevity is a by-product of adaptation through hitchhiking. We adopted a population genetic approach to explain the ability of natural selection to modulate lifespan in animals.

### Damage-Centric and Imperfectness Model of Aging

These series of theories emphasize that the basis of aging is accumulated molecular damage (Medawar, 1952; Harman, 1956). For example, reactive oxygen species (ROS), free radicals, and other oxidants lead to damage to DNA, proteins, and other molecules in the cell. ROS and other metabolic wastes are deleterious products generated at varying amounts that mostly correspond to the metabolic activities and energy expenditure of organisms. High metabolic rate and energy expenditure, as seen in flight, should produce enough damage capable of driving rapid senescence and leading to short lifespan. This hypothesis does not hold for birds and bats. Despite having a metabolic rate, about twofold more than mammals of comparable size, birds with active flight do not show signs of rapid senescence and have a maximum lifespan that triples that of similar-sized mammals reared in captivity (Holmes and Austad, 1994; Munshi-South and Wilkinson, 2010). Birds appear to have evolve mechanisms to protect against the deleterious effect of high metabolism. ROS and other free radicals undoubtedly induce damage and oxidative stress, but this, by itself, is not sufficient to drive aging. Consistent with this idea, there is no evidence of some special antioxidant defense, as would have been expected, in extremely long-lived animals compared with short-lived species (Andziak et al., 2006). It is reasonable to assume that this oxidative damage, nevertheless, contributes to the overall aging progress (Shi et al., 2019).

The imperfectness model, however, offers a mechanism that describes aging as a consequence of largely non-random damage that accrues due to the imperfect nature of biological systems (Gladyshev, 2013). An organism encompasses different organs and cell types, the genome, proteins, signaling networks, macro-molecules, as well as the rest of the cellular machinery, and collectively, they carry out the physiological and metabolic activities required for optimal system function. These physiological functions unavoidably produce numerous forms of damage collectively known as the deleteriome (Gladyshev, 2016). When this damage is generated in organisms with fully differentiated non-renewable cells and structures, it will accumulate and cause aging. As the processes that generate these damages are under genetic regulation, different species will generate damage according to their genetic programs. In that sense, there is a nice connection between the deleteriome model of aging and the adaptive hitchhiking model. Indeed, control of lifespan involves explanations about the cause of variation in lifespan across the clades of organisms and how it can be fixed. All organisms generate damage and also develop mechanisms that protect against this damage. The regulation of biological processes and pathways in extremely long-lived animals (Moreno Santillán et al., 2021; Wilkinson et al., 2021) shows patterns of adaptation that are distinct from those of short-lived animals, and this is reflected in the damage that they produce (Bundgaard et al., 2019; Kacprzyk et al., 2021). Accordingly, modulation of related pathways in short-lived species is known to extend the lifespan (Leiser et al., 2013). Therefore, similar to the deleteriome model, wherein cumulative damage varies across taxa and contributes to variation in the aging process, we posit that control of lifespan is adaptive and driven by genetic heterogeneity.

### Evolutionary Theory of Aging

Antagonistic pleiotropy and the disposable soma theories of aging are related in that they emphasize a tradeoff between reproductive fitness and extended lifespan. Intrinsically, tradeoffs are rampant in the wild and are usually biased toward species fecundity, which is expected, especially in a volatile environment or when self-perpetuation becomes increasingly difficult (Reznick et al., 2006). This bias has been described among vertebrates (Marshall et al., 2017) and invertebrates (Snell and King, 1977), both in the wild and under controlled laboratory environments. More so, antagonistic pleiotropic phenomenon seems to be pervasive in the genome (Austad and Hoffman, 2018). One such example is the mTOR and insulin signaling pathways that are responsible for early-life development but contribute to the aging process. Whereas our model is not antagonistic, it could be likened to pleiotropy in that selective pressure acting at a locus influences both the adaptability and longevity of an organism. Moreover, our model does not assume that tradeoff should exist between reproduction and longevity as no genetic evidence of resource allocation and redistribution exists between somatic maintenance and fecundity; instead, we posit that longevity hitchhike along with adaptation, which, in turn, can contribute to overall reproductive success. It should be noted, however, that both models may apply to regulate lifespan.

### The Pleiotropy Model of Aging and Cancer

The pleiotropy phenomenon is widespread in living organisms whereby multiple phenotypic traits could be attributed to a single locus. The protagonist pleiotropy of chromosomal damage model proposed two major forms of damage resulting from spontaneous DNA mutations; such damages could lead either to cancer or non-cancer pathologies (de Grey, 2007). It posits that the stronger the evolutionary pressure to prevent cancer-related deaths is the less likely the aging, non-cancer-related pathologies are. Thus, the selection force against the onset of cancer through the DNA maintenance machinery directly prevents non-cancer forms of aging. This is a cancer-resistance mechanism that indirectly promotes longevity; it has actually been shown by several studies that an underlying tradeoff exists between cancer and aging, such as the canonical p53 pathway (Tyner et al., 2002; Yashin et al., 2009). In line with our hitchhiking model, however, selection could act on non-cancer-related loci to extend lifespan due to selection pressure at the loci with linkage disequilibrium. Hence, it is highly likely that evolutionary constraints acting to prevent cancer also act against aging-related pathologies. However, the source of selection that extends lifespan or causes slow aging is not expected to originate solely from the selection signal that prevents cancer-causing mutations, but it is more likely that ecological adaptation and genetic heterogeneity play significant roles as well. Natural selection is, by itself, restrained by the randomness of DNA mutations, gene variants, and the pleiotropic interaction that exists between loci or genes, which is the basis of the antagonistic pleiotropy hypothesis (that evolution could adopt a pleiotropic trade-off of longevity for reproductive fitness). Under the strict rule of antagonistic pleiotropy, it is unlikely that lifespan extension would be achieved in the certainty that post-reproduction would be marked by deleterious manifestation of mutations. However, under positive pleiotropy, selection for early-life fitness could contribute to post-reproductive fitness and extend lifespan (Maklakov et al., 2015). Therefore, it is probable that a mutation could attain fixation that confers early life fitness and extends lifespan without tradeoff as observed in Drosophila and nematodes (Chen and Maklakov, 2012; Khazaeli and Curtsinger, 2013; Kimber and Chippindale, 2013). Altogether, we infer that pleiotropy is widespread in the genome and plays an important role in moderating species fitness through selection.

## Concluding Comments

Exceptionally long-lived mammals and other vertebrate species might have acquired longevity traits over millions of years; they have independently overcome ecological constraints through adaptive mechanisms that concomitantly promote their longevity. The pattern of the aging process and longevity observed across the tree of life varies greatly and is largely maintained by the force of natural selection acting on species genome under given environment. Adaptive evolution is a non-random process that is dependent on random mutations in the genome; this could explain the importance of suitable genetic variants to drive lifespan extension, which might not be compatible with fitness or adaptability in short-lived species. Our model, in the context of population genetic framework, explains the mechanism through which extended lifespan evolved and is maintained in long-lived animals. The hitchhike model is an approach that is centered around animal adaptation driven by natural selection. We anticipate that the adaptive hitchhike model would generate many studies that would identify genomic elements under natural selection that drive extended lifespan in target species.

## Author Contributions

XZ conceived the idea. OO drafted the manuscript. XZ, VNG, and OO edited the draft. All authors approved the final manuscript.

## Conflict of Interest

The authors declare that the research was conducted in the absence of any commercial or financial relationships that could be construed as a potential conflict of interest. The handling editor declared a past co-authorship with one of the authors VNG.

## Publisher’s Note

All claims expressed in this article are solely those of the authors and do not necessarily represent those of their affiliated organizations, or those of the publisher, the editors and the reviewers. Any product that may be evaluated in this article, or claim that may be made by its manufacturer, is not guaranteed or endorsed by the publisher.
